# Neurofeminism and feminist neurosciences: a critical review of contemporary brain research

**DOI:** 10.3389/fnhum.2014.00546

**Published:** 2014-07-25

**Authors:** Sigrid Schmitz, Grit Höppner

**Affiliations:** Faculty of Social Sciences, Chair of Gender Studies, University of ViennaVienna, Austria

**Keywords:** feminist neurosciences, neurosexism, neurofeminism, neuro-epistemologies, neurocultures, neuropedagogy

## Abstract

To date, feminist approaches to neurosciences have evaluated the debates surrounding practices of knowledge production within and research results of contemporary brain research. Consequently, neurofeminist scholars have critically examined gendered impacts of neuroscientific research. Feminist neuroscientists also develop research approaches for a more gender-appropriate neuroscientific research on several levels. Based on neurofeminist critique feminist neuroscientists aim to enrich neuroscientific work by offering methodological suggestions for a more differentiated setup of categories and experimental designs, for reflective result presentations and interpretations as well as for the analysis of result validity. Reframing neuro-epistemologies by including plasticity concepts works to uncover social influences on the gendered development of the brain and of behavior. More recently, critical work on contemporary neurocultures has highlighted the entanglements of neuroscientific research within society and the implications of ‘neurofacts’ for gendered cultural symbolisms, social practices, and power relations. Not least, neurofeminism critically analyses the portrayal of neuro-knowledge in popular media. This article presents on overview on neurofeminist debates and on current approaches of feminist neurosciences. The authors conclude their review by calling for a more gender-appropriate research approach that takes into account both its situatedness and reflections on the neuroscientific agenda, but also questions neurofeminist discourse in regards to uses and misuses of its concepts.

## Neurosexism, neurofeminism and feminist neurosciences: a ‘herstorical’ narrative

By the middle of the 1980s–and starting with Fausto-Sterling (1992) book on *Myth of Gender* at the latest–feminist science studies have critically examined the knowledge production in the neurosciences and its impacts in gendered significations, gender roles and social gender relations.^1^ Scholars of the humanities, of the social and cultural studies, and from neuroscience itself have critically examined theoretical, methodological and methodical neuroscientific approaches. Scholars of feminist science studies have elaborated a long-standing critique of neuroscientific empirical research and underlying theoretical concepts, claiming that they present a seemingly undisturbed history of science and its actors from a positivist perspective of enlightenment. In this paper, we present an overview of recent approaches to critically reflect these problematic narratives in different ways. We will present a ‘herstory’ rather than a ‘history’ when referencing the wide scope of research from colleagues who have evaluated the findings, methodologies, and processes of knowledge production in the neurosciences that are all situated within social contexts. These studies have outlined the gendered constructions within research as well as the impacts of references to supposed neuroscientific “facts of the sexed brain” on various levels. Critical gender, feminist, and queer scholars have developed a term for these uncritical biases in research and public perception, and for their societal impacts on the individual, structural, and symbolic level: *neurosexism* (Fine, 2010). Their outstanding scholarly work has explored such biases in neuroscientific research for more than three decades now.

As a counterpoint to neurosexism, and following (Bluhm et al., 2012), we use the term *neurofeminism* to encompass this work that evaluates practices of knowledge production within neuroscience. Neurofeminism critically validates gendered assumptions of contemporary brain research and examines the impacts of references to neuroscientific research on social gendered order and cultural significations. In consequence, neurofeminism circumscribes the analytical perspective for re-evaluating methodological constraints and knowledge productions within the neurosciences. Based on a bio-cultural perspective, neurofeminism additionally highlights the inseparable entanglements between the development of biological matter and social influences.

Neurofeminist scholars not only aim to critically examine neuroscientific knowledge production but are also engaged in developing differentiated approaches for a more gender adequate neuroscientific research. The latter approaches, subsumed as *feminist neurosciences*, develop assessment tools for multiple participant categorizations (Joel et al., 2013), draft non-linear experimental designs and processes of data analysis (e.g., Dussauge, 2014; Kaiser, 2014) and seek for alternative models of non-generalized interpretations, which are based upon intersected categories such as gender, ‘race’ and age (e.g., Kuria, 2014). Feminist neuroscientists generally seek to elaborate the relation between gender and the brain beyond biological determinism but still engaging with the materiality of the brain. They aim at a more differentiated setup of categories and experimental designs, getting more transparency in the constructive processes of result presentations and interpretations.

In the following, we outline the approaches and findings of the NeuroGender Network (in Section The NeuroGender Network), an expert group that has been founded in 2010 within the scope of neurofeminist agenda. We then elaborate our ‘herstory’ in Section Concepts, Knowledge Productions, and Reflective Approaches of Feminist Neurosciences by summarizing findings of recent critical research on sex/gender and the brain. We incorporate different perspectives of neuroscientific epistemologies that highlight plasticity concepts in particular in order to gain a more differentiated view on brain-behavior development in gendered socio-cultural experiences and contexts. In Section Neuro-epistemologies, Neurocultures and Their Impacts, we focus on current neurocultural discourses, their references to neuroscience and, vice versa, the impacts of neuroscientific knowledge production on gendered social power relations. Finally, we also question the neurofeminist discourse in regards to uses and misuses of its concepts. To point to the narrative structures of ‘neuroscientific stories’ we decided to put terms that—in our view—embed constructive meanings in single quotation marks, though these terms may be treated as ‘facts’ in scientific and popular debates. This format may irritate the reader, but the irritation is deliberate.

## The neurogender network

In March 2010, the Center for Gender Research at Uppsala University, and Isabelle Dussauge and Anelis Kaiser in particular, launched the first international and transdisciplinary NeuroGenderings conference in order to make available a platform for the exchange between scholars of neurofeminism. This conference was funded by the Swedish Research Council, as part of the excellence program *GenNa: Nature/Culture and Transgressive Encounters*, and by the Body/Embodiment Group at the Center for Gender Research.^2^ At this meeting, the NeuroGenderings Network^3^ was initiated. Scholars from Europe, the United States, Canada, and Australia represent a broad range of disciplines such as neuroscience, the humanities, social and cultural studies, gender and queer studies, feminist science studies, and science and technology studies. They all research a variety of issues in the field of gender and the brain, evaluate the current state of neuroscientific methods, findings, representations, and interpretations of empirical brain research (neurofeminism), initiate dialogue across disciplinary borders, and develop detailed and enriched approaches for neuroscientific analyses themselves (feminist neuroscience). Moreover, the NeuroGender Group aims to develop concepts for more reflective debates in education and in all social spheres (an approach we call *neuropedagogies*). The NeuroGenderings Expert Group published its first results in a special issue of the journal *Neuroethics*, entitled “Neuroethics and Gender” (for an overview, see Dussauge and Kaiser, 2012b).

The network grew after its second conference, entitled ‘NeuroCultures—NeuroGenderings II’, which was held at the University of Vienna from 13–15 September, 2012.^4^ The NeuroGender experts discussed the impacts of neuroscientific research on gender constructions in socio-political and cultural fields and–vice versa–analyzed the social and political underpinnings of the ongoing cerebralization of human life. The recently published volume *Gendered Neurocultures: Feminist and Queer Perspectives on Current Brain Discourses* (Schmitz and Höppner, 2014) brings together differentiated analyses of scientific knowledge production focusing on sex, gender, and the brain and offers insight into gendered norms that frame current neurocultures. It also demonstrates how some of these norms could possibly be transformed while revealing how others persist in scientific and popular discourse. The volume, finally, presents novel concepts for incorporating gender-appropriate neuro-pedagogies in teaching and social discourse.

The NeuroGender Expert Group is in itself not homogeneous, neither in its disciplinary connection to the field, its perspective on neurosciences and current neurocultures nor concerning the theoretical assumptions and conclusions about these issues. In consequence, though ‘our’ knowledge production (as all scientific knowledge productions) has some common lines, others are more controversial (Kraus, 2012b). The NeuroGenderings III conference, May 8–10, 2014 in Lausanne (Switzerland),^5^ organized by Cynthia Kraus and Anelis Kaiser, highlighted the different standpoints in the debate, and the discussion proceeds.

## Concepts, knowledge productions, and reflective approaches of feminist neurosciences

Neuroscience is currently a key area of research regarding the question of what constitutes the human (for overview, see Littlefield and Johnson, 2012). With its modern methods of brain imaging and thus the apparent ability to see into the living brain at work, structures and activity networks appear to be precisely localizable (e.g., Rose, 2005; Fitzpatrick, 2012). Until today, neurosciences and even more popular science media (cf. Joyce, 2005) take behavior, thinking, and acting often as being explainable and even predictable via these biological materialities. Particularly neurodeterminist notions of a ‘sexed brain’ are being transported into public discourse (e.g., Pease and Pease, 2004; Cahill, 2005; Brizendine, 2007, 2011; Gray, 2012) without reflecting the biases in empirical work.

The basic assumption of two sexes is the premise for applying difference-oriented methods in brain research, through which each group is assumed to be inherently homogenous. However, the outcome of research on differences between women and men in terms of linguistic abilities, spatial orientation, or mathematics—that is, of cognitive capacities in general—is by no means conclusive (Schmitz, 1999; Coluccia and Louse, 2004; Spelke, 2005; Mehl et al., 2007; Else-Quest et al., 2010; Lavenex and Lavenex, 2010; Fausto-Sterling et al., 2012a,b); neither are results on emotional or rational processing (Karafyllis, 2008), nor is the state of the art on brain basics for sexual orientation and desire. The influence of heteronormative notions of sexual orientation and desire to sex/gender determinism has been impressively shown, for example, by Dussauge and Kaiser (2012a).

In meta-analytic reviews, Janet Hyde emphasized more behavioral similarities than differences between women and men (Hyde, 2005), gender similarities have been shown impressively concerning math performance (Hyde and Linn, 2006; Hyde et al., 2008). Feminist neuroscientists have uncovered inconsistent findings concerning sex differences and elaborated similarities between or variations within the gender groups, not only on the level of behavior and performance but also concerning their apparently biological sources, i.e., the brain networks and their functions (Frost et al., 1999; Blanch et al., 2004; Ulshöfer, 2008; Wallentin, 2009; Jordan-Young, 2010; Bluhm et al., 2012; Jordan-Young and Rumiati, 2012; Kuria, 2012; Roy, 2012; Vidal, 2012; Dussauge, 2014; Kaiser, 2014; Sommer et al., 2004, 2008).

Approaches questioning the boundaries between sex and gender prompted us to use the term *sex/gender* in reference to analyses of neuroscientific research. According to feminist science studies, sex is not a purely physical or material fact but is deeply interwoven with social and cultural constructions (Fausto-Sterling, 2012). Following this concept, the term *sex/gender* (first applied to the neuroscientific context by Kaiser et al. (2009) is deliberately used throughout this paper to emphasize the inextricable entanglements of both categories in a bio-cultural approach. Recently, the importance of including intersected categories into neuroscientific research has been highlighted (e.g., Kaiser, 2012; Hyde, 2014). Intersectional perspectives help to outline the entanglements of categories in neuroscientific research, for example, when racism and ageism are connected with gender. Kuria (2014) elaborates how ‘race’/ism is implemented in empirical neuroscientific knowledge production on sex/gender. In her analysis, she uses a medical history perspective and the psychoanalytical work of Grada Kilomba to expose the processes of knowledge production as an “act of decolonizing knowledge” (p. 110). Not only does Kuria show the relevance of integrating ‘race’ as an analytical category when analyzing neuroscientific work and its inherent gender differences, moreover she reminds the NeuroGender network itself to reflect on its own ‘white’ norms as aspects that call for critical analysis. Taking a focus on current brain-imaging research of facial affect, Gumy (2014) elaborates intersections of age and gender. She evaluates which fMRI tools allow a significant difference to be seen in brain activation between adolescents and adults. Gumy argues that current concepts of the “cerebralization of adolescence” (p. 258) itself produce differences between boys and girls as neuroscientific scholars never understood the brain as being unisex but rather pre-defined it as male-connoted. In consequence, as Gumy argues, sex/gender is acting performatively in the construction of the ‘adolescent brain’ on various levels. By presenting neurofeminist empirical critiques and aiming for a more gender-appropriate research, these approaches focus on important methodological aspects and biases in the construction of sex/gender, ‘race’ and age in brain research.

Differentiated analyses systematically investigated methodical influences on neuroscientific knowledge production in sex/gender research. Scholars such as Bishop and Wahlstein (1997), Fine (2013), Kaiser et al. (2009) and Schmitz (2010) have uncovered variations in data selection, statistical analyses, and computer tomographic calculations that contradict generalizations being made throughout various studies. The fact that these meta-studies have been included in scholarly neuroscience journals attests an increasing sensitization towards critical reflections on methods within brain analyses. Nonetheless, even now, studies that establish differences between sex/gender are published more often than studies that do not find differences (Kaiser, 2012; Fine, 2013). Although the reasons of such ‘publication biases’ may be manifold (e.g., caused by the emphasis for getting research funding within the sciences or due to catching the interests of the public target groups in popular press), they still manifest notions of a binary gender order and foster the persistence of seemingly biologically determined gendered significations. Even more rigid gendered stereotypes endure, particularly when it comes to the transgression of ‘scientific knowledge’ into the public discourse and when addressing social issues within popular media. For example, recent in-depth analyses have evaluated the scope of neuroscientific knowledge *and* examined the media discourse surrounding the debates on the function and transformation of the hormone oxytocin. In French media, oxytocin was recently referred to as a ‘maternal’ hormone (Fillod, 2014) or, in public discourse, even as the ‘cuddle’ hormone. Though oxytocin has always been regarded as a female hormone, it began being treated as *the* biological basis for pair bonding and trust (Matusall, 2014), despite the fact that there are no neuroscientific findings to support this claim. Odile Fillod and Svenja Matusall showed how media articles selectively choose the one or the other ‘scientific fact’ (without reflecting on the inconsistencies and the lack of valid findings) to legitimate social gender relations of ‘motherhood’.

Brain images of brilliantly colored activation areas present local distinctions and precise areas of processing in line with behavioral and cognitive performances. These visualizations are often taken as evidence of what is inside (or outside) of the brain (McCabe and Castel, 2008). They serve as a metaphoric concept of science that proposes discovering and representing ‘nature as it is’. Beside an abundance of critical analyses on the processes of brain image constructions (Beaulieu, 2002; Gallagher, 2011; Fitsch, 2012; for an overview, see Choudhury and Slaby, 2011), nevertheless, neuroscientists are well aware of both the constructive nature of these images (Beaulieu, 2001; Joyce, 2005) and the mutual biases in neuroscientific research (Vul et al., 2009; Henrich et al., 2010). Brain images are by no means direct portrayals of the interior of the brain. The transformation from corporeal matter to non-corporeal data and conversion back to an image of a brain’s matter (Schmitz, 2003) is certainly not unprecedented. In terms of laboratory studies, particular “inscriptions” (Latour and Woolgar, 1979) are well in line with constructive processes of brain imaging. In consequence, brain image construction always depends on the context in and on the decisions with which the images are produced. These decisions depend, for example, on the theoretical approach of the analysis, on the methods applied during the experiment, on negotiation processes within a lab, on research aims, or on the conscious and unconscious understandings and beliefs of researchers, to name only a few intervening factors (e.g., Beaulieu, 2001). However, this is not to say that knowledge of the brain that has been gained through image technology is random or inapplicable. On the contrary, and especially for biomedicine, ways of constructing knowledge through specialized procedures are useful for different diagnostic fields, therapy, or neurosurgery. Nevertheless, brain-imaging procedures are still constructions with particular aims and functions, highlighting the one brain area or the other vessel structure, subtracting one activity from another.

The analyses of critical neuroscience are also of importance for reflective approaches to the gendering of brain images. Recent analyses have shown that due to the selection of calculation processes gender differences or a lack of those differences can appear in images of the same experimental group (Kaiser et al., 2007; Fitsch, 2012, 2014; Nikoleyczik, 2012; Maibom and Bluhm, 2014). As brain images that are presented in scientific papers include the decisions made by researchers and research groups while computing an image, these studies should include transparency of their categories and calculation processes in data analysis. The use of brain images becomes problematic if they are presented out of context in order to make generalizing statements about predefined groups based on gender, age, ethnicity, or other universalized categories (Schmitz, 2010; Gumy, 2014; Kaiser, 2014; Kuria, 2014). It turns out to be even more problematic if brain images are used in popular science publications (in popular media as well as in popular books) to transport simplified statements about group differences in brain and behavior or even predictions about further behavior of seemingly homogenized groups (e.g., Pease and Pease, 2004; Cahill, 2005; Brizendine, 2007, 2011; Gray, 2012). Scholars from neurofeminism have elaborated the transgression processes of apparent biological explanations that are taken for granted and are transformed in public discourse to confirm supposedly rigid sex differences—irrespective of whether gender differences are constituted in gendered social orders or not (Vidal and Benoit-Browaeys, 2005; Vidal, 2005, 2012; Fillod, 2014; Jordan-Young, 2014). These analyses also show how the newest scientific findings are referenced in order to serve the legitimization of social hierarchies, inclusions, and exclusions (Höppner and Schmitz, 2014; O’Connell, 2014).

Finally, and with respect to the conceptual background of neuroscientific research, a differentiated understanding of sex/gender and the brain was developed in the context of plasticity concepts in the neurosciences, the history of which spans more than 50 years, beginning with the first notions of structural changes in synaptic connections, developed in animal studies in the 1960s (Rosenzweig et al., 1962). Findings from animal studies led to the conclusion that the processing of environmental stimuli already prenatally effects the development, decomposition, and alteration of synapses between neurons. Learning experience stabilizes and destabilizes the central nervous network along its situatedness in a particular environmental context to which it is supposed (Hubel and Wiesel, 1970). The concept of brain plasticity points out that brain structures and brain functions are not in any shape or form determined by evolution or remain unchanged during a life span. At birth, the brain is not at all branded or defined, and this network of nerve cells, neuronal fibers and their synapses is not ‘completely formed’ by genetic information. As Vidal (2012, p. 297) puts it: “The human brain is made up of 100 billion neurons and 1 million billion synapses which are the junctions between neurons, while there are only 6000 genes involved in the nervous system. This means that there are not enough genes to control the building of our billions of synapses”.

This concept of brain plasticity and the brain’s ability to adapt to its environmental influences applies to all areas of the brain, but outstanding to the most complex networks of the cortex. As a result, neuronal networks ‘learn’ repetitive patterns of information and embody them structurally and functionally. Neuroplasticity studies in the 1980s and 1990s have pointed to the adaptive potential of sensory and motor areas in primates (e.g., Jenkins and Merzenich, 1987; Kaas et al., 1990) and in the last decades plasticity in the human cortex is in the center of a whole scope of analyses (see below). Environmentally influenced neural and synaptic plasticity, down to physiological regulations and gene expression (for an overview, see Kandel et al., 2000), are taken as the cellular and molecular basics for learning principles in the central nervous system. The alteration of neuronal networks is a lifelong process, which occurs through experience and with every learning activity. Brain plasticity is not only the basis for learning over the entire lifetime, but is also necessary for brain functions. The tremendous dynamic ‘nature’ of brain plasticity, particularly in humans, this constant interplay between the outside environment and biological structure, is considered as a decisive advantage for human cognitive abilities.

Today, brain plasticity studies in humans address the development of different language networks and brain functionalities in line with individual language biographies (Bloch et al., 2009), changes in spatial and motoric areas correlated to long-term navigation experience (Maguire et al., 2000) or structural changes in the corpus callosum (e.g., Gaser and Schlaug, 2003) and motoric brain networks following shorter periods of learning juggling (Draganski et al., 2004). Plasticity concepts have therefore led to a redefinition of cause and effect in ‘neuro-argumentations’, as the biological system of the brain is extremely open and able to adapt diverse influences over the course of a lifetime. From this constructivist perspective, the brain cannot be characterized as solely physical matter and as the only essence of behavior. It must be analyzed in regards to its continuous entanglements with the outer world. Cultural and social experiences influence behavior by forming and shaping the biology of the brain. Recent studies on how the experience with virtual games diminishes sex/gender differences are indicative of the necessity of these constructions of competences (Feng et al., 2007), for which brain studies have also argued (Jordan-Young, 2010). During the last decade, neurofeminist scholars more and more used the perspective of plasticity to analyze the material-discursive entanglements of brains and their environments (Schmitz, 2010; Jordan-Young and Rumiati, 2012; Vidal, 2012).

Accordingly, brain images are snapshots of a certain moment of physical materiality, which is always connected to individual biographies. Results of brain scans can thus not provide information on the processes that led to these developments, neither from nature nor from culture. Despite this obvious critique, the “*snapshot approach”* is mostly used in place of plasticity considerations for the interpretation of results (Schmitz, 2010), and it persists even in recent sex/gender-related brain imaging studies, as Fine (2013) has analyzed.

Neurofeminist analyses also investigate the stereotype thread and its consequences. The overtaking of gendered stereotypes into self-schemata impacts cognitive performance, behavior, and even neuronal processes (Spencer et al., 1999; Massa et al., 2005; for an overview, see also Fine, 2010). Taking the example of spatial orientation, a prominent issue when it comes to determining sex differences in behavior, the mutual influences on the development of cognitive strategies have already been elaborated (Schmitz, 1997, 1999) and their impacts on brain development have been discussed intensively (Fine, 2010; Jordan-Young, 2010).

In sum, neurofeminism has developed a theoretical and methodological agenda to critically review neuroscientific knowledge production concerning sex/gender aspects. Differentiated analyses help to foreground diversities and dynamic processes in place of binary gender categories (for an overview, see the collection on *Neurofeminism: Issues at the Intersection of Feminist Theory and Cognitive Science* edited by Bluhm et al., 2012, the special issue on *Neuroscience and Sex/Gender* edited by Dussauge and Kaiser, 2012b, and the anthology on *Gendered Neurocultures: Feminist and Queer Perspectives on Current Brain Discourses* edited by Schmitz and Höppner, 2014). Scholars of the NeuroGenderings Network, however, aim for more than only critical evaluation of existing research. From feminist and queer perspectives, they reflect the influences of gendered and intersected norms and values in brain research and brain imaging procedures and, conversely, research the impacts of neuroscientific knowledge production on processes of normalization (Dussauge, 2014; Fitsch, 2014; Kaiser, 2014; Kuria, 2014). Moreover, feminist neuroscientists aim to enrich neuroscientific research by considering perspectives, which have found only less attention in such approaches up to date, for example, the development of assessment tolls for the multiple dimensions of participant categorizations (Joel et al., 2013).

## Neuro-epistemologies, neurocultures and their impacts

The brain plasticity concept is important for deconstructing unilinear statements about a supposedly biological determination of behavior, attitudes, etc. In narrating plasticity stories, neurofeminist scholars mainly stress a return of genealogies of cause and effect, arguing that gendered social experiences and power relations impact the forming of the gendered brain’s structure and function more than vice versa (e.g., Vidal, 2012). But the use of the brain plasticity concept—albeit extremely useful for pointing out the inscription and embodiment of individual experiences, social structures, and cultural norms—also challenges neurofeminist discourse in two ways. Firstly, brain plasticity analyses follow an account that refers to the discursive forming of materiality and thus emphasize the meaning-making processes inscribed in the brain (Schmitz, 2014a). By pointing solely in this direction, they do not take into account the brain’s agency. With reference to plasticity concepts, the materiality of the brain remains to be framed as a more or less passive reactor to the attribution of gendered (and intersected) significations. The perspective of a feminist materialist approach (e.g., Barad, 2003; Alaimo and Hekman, 2008; Dolphijn and van der Tuin, 2012) points to brain plasticity rather as an intra-active phenomenon. In terms of Karen Barad’s onto-epistemological framework, this perspective particularly addresses the dynamic processes that constitute ‘real’ world phenomena between material and meaning-making (discursive) components (Barad, 2007). Based on this framing, neuro-materiality can be discussed in terms of culture, society, cognition, and behavior, which all give meaning to each other in developmental processes of enacting and intra-acting. If brain and culture are understood as being indivisibly intertwined in an assemblage of reciprocal exchange, constituting and continuously re-shaping each other, then the phenomenon of the ‘brain-body-in-culture’ not only passively awaits its shaping and forming from the outside world. Hence, the disclosure of the impartible entanglements of brain, bodies, mind, behavior, socio-cultural contexts, and meaning-making serves as an inspiration for neurofeminist debates. Wilson (1998) already started employing these approaches in *Neural Geographies* and scholars from the NeuroGender Network have recently called for addressing the brain’s agency in a non-essentialist manner (Dussauge and Kaiser, 2012a; Kraus, 2012a; Schmitz, 2014a).

In consequence, differentiated approaches to gender issues in neuroscience also have to take into account the dynamic constitutions of the brain sex/gender network, embedded in biological, psychosocial, and sociocultural intra-actions. By debating concepts of current brain research and by theoretically and empirically discussing their consequences (e.g., Fine, 2012; Joel, 2014; Maibom and Bluhm, 2014; Pitts-Taylor, 2014; Roy, 2014), a reframing of neuro-epistemologies should allow for questions as to how concepts (even one’s own) and discourses can be read against the backdrop of the feminist debate surrounding materialism (Schmitz, 2014a,b,c). Do neurofeminist concepts grasp corporeal materiality beyond essentialist determinism and ontological entities? To what extent do these concepts take the conscious and non-conscious agency of materiality seriously? Do they consider the entanglements and the intra-acting material-discursive components that mark each other by ascribing meaning to materiality within a particular context? Additionally, does scientific and popular discourse question the hybrid conceptions of brain-bodies-in-cultures in terms of their potentials for disrupting nature–culture dichotomies on both material and epistemological levels? Or do the limitations and dichotomist conceptions continue to prevail? Last but not least, does the feminist materialist framework, which highlights the mutual intra-actions of neuro-cultural phenomena in particular, cover all the important aspects in this discourse or does it also challenge the feminist self-conception of political science (a ‘dissensus’ question to which we will refer in the last section of this paper)?

Secondly, merely turning around the cause–effect genealogy does not prevent the use of essentialist concepts. The knowledge production and scientific framework that are elaborated in the field of the neurosciences both form the reference point for a wide scope of other disciplines and contemporary academic discourses (cf. Littlefield and Johnson, 2012). Not only science and psychology but also education, economics, sociology, the humanities, and philosophy refer to the results of brain research to explain their concepts of societal processes. They all treat the individual as a “cerebral subject” (Ortega and Vidal, 2007)—the anthropological figure of the human according to which the brain constitutes the self. Current neurocultures comprise the conceptions of how thought, behavior, subjectivity, and identity should correspond to the brain’s biology and one could thus go so far as to claim that neurocultures represent a new paradigm—for research as well as for social discourse.

Neurocultures are based on the development of an all-explanatory epistemology of the brain (Koslow, 2000), an endeavor that started in the 1990s with the “Decade of the Brain” and the first “Human Brain Project” (OHBM, 2001). This research agenda was sponsored by the US National Academy of Health during George W. Bush’s presidency, and is currently being followed up by the new U.S. BRAIN Initiative,^6^ the EU-based “Human Brain Project”^7^ and the Swiss “Blue Brain Project”.^8^ The latter project is particularly geared toward building an artificial brain computer. The wordings of these projects are full of metaphoric pictures: “adventure”, “discovery”, “enlightenment”. They thus follow, once again, a metaphysical approach to science, reaching out for an all-explaining knowledge framework with which to explain ‘the human’ (including and referring also to animal studies). This framework is reminiscent of the ‘promises’ of bio-technologies that Donna Haraway already elaborated decades ago (Haraway, 1992). These developments recall the need for a critical approach to researching and uncovering the impacts of neurocultural references that legitimize social structures and even gendered relations: especially the fact that political interventions are also legitimized using these references should inspire critical neurogender analyses. Schmitz, for example, has analyzed the specific argumentation of “modern neurodeterminism”, a crucial aspect within these debates. Modern neurodeterminism does not care whether brain structures and functions are innate or formed by experience, considering it to be irrelevant whether the individual brain is formed by nature or nurture. Nevertheless, these paradigms remain based on essentialist concepts of the brain. Brain materiality and functionality are believed to be essential for explaining and even predicting the outcome of behavior at the moment of measurement (for a detailed development of this concept see Schmitz, 2012).

There is an interesting ambivalence that is not articulated in current neurocultural discourse: while plasticity concepts are included in modern neurodeterminism up to the moment of measuring, they are dropped again when it comes to predicting future behavior. Although insisting on the forming of biological materiality from ‘outside’, neurocultural discourse is in danger of remaining connected to concepts that presume to predict behavior, thinking, and acting due to biological entities from ‘inside’. The impacts of these normative framings on cultural understandings, social practices, and governmental discourses are already the subject of critical analyses (Choudhury et al., 2009; Choudhury and Slaby, 2011). Neurofeminism must question the gender-based and intersected legitimations that are drawn from neurocultural discourse and practices, in particular because neuroscientific knowledge production can be used for various socio-political in- and exclusions (O’Connell, 2014).

One example of a critical examination of neurocultures is Höppner and Schmitz (2014) analysis which pursues the question of how the phenomenon of neuropharmacological enhancement is discussed in the German media. The analysis of 21 public media articles (published between 2006 and 2011 in four German online journals) shows that self-optimization of the brain with the help of neuropharmaceuticals is increasingly predicted as a universal strategy for success. Whereas success-oriented males tend to aim for the improvement of their rational skills, success-oriented-women should focus on regulating their self-confidence. Moreover, the articles once again manifest a hierarchized status quo of rational skills over emotional capacities and a different proficiency level that neuro-enhanced subjects could achieve depending on their gender. While women would need a continual consumption of neuro-enhancers in order to achieve a proficiency level similar to male capacities for a limited time, men should take neuro-enhancers only once a while to selectively enhance their supposedly high capacities (a result of their biological setup) to become the best within the group of the best men. In contrast, a continual consumption of Ritalin by women is shaped as resulting in distress and negative reactions from the social environment. This analysis illustrates that the paradigm of pharmacological neuroenhancement is not free of gendered implications in a success-oriented society but rather reproduces biologist significations of women and men. Within the current popular debate, what stands out in particular is the paradigm of individualization and self-responsibility in neoliberal society. Equal opportunities qua the optimization of brains are addressed in the sense of ‘everyone is his/her own fortune’. This falls in line with a disregard for social structures that perpetuate gendered discriminations on the labor market. Central to this logic is the individualization of deficiency concepts and the call for self-responsibility to improve one’s own brain for seemingly personal needs, instead of fighting social causes of gender inequality. The possibility of achieving equality appears as a solely neutral endeavor.

In the modern meritocracy, the plastic brain is used as the starting point for modulating and optimizing human behavior (Maasen and Sutter, 2007; Pitts-Taylor, 2010). Understanding the brain as an instrument that is open for manipulation and modulation, which should be enhanced by using the most diverse technologies, these neuro-optimizations intervene in the body on a profound level and thus co-constitute the cerebral subject (Schmitz, 2012). Brain vocabulary produces a culturally and historically specific version of the human being and, as such, impacts individual, social, cultural, and political spheres. As education, social, and cultural studies as well as philosophical disciplines refer to the results of brain research and turn the brain into the central category of the cerebral subject, definitions of the self, social processes, or the idea of a future humanity are debated in terms of neuro-argumentation (Vidal, 2009). Neuro-argumentation emerges as the central figure in configurations of the “politics of life itself” (Rose, 2007), in the emergence of neuro-governmentalities (Rose, 2005; Maloni, 2010; Rose and Rose, 2013), in new framings of biological citizenship (Rose and Novas, 2005) and in the “management of the mind” (Rose and Abi-Rached, 2013). It should be an urgent task for gender and neurofeminist research to reassess and reflect upon these conceptions (Schaper-Rinkel, 2012). How are the concepts of brain plasticity taken up in times of neoliberalism and how do they affect the connected neuroscientific discourses on gendered concepts (Pickersgill, 2013; Höppner and Schmitz, 2014; Schmitz, 2014b)? Do these new concepts carry emancipatory potential for gendered positions and possibilities of action, or (how) do they retransmit gender binaries and boundaries?

Last but not least, and following the goal of improving neuroscientific knowledge production, it is necessary to reflect both the scientific concepts/methodologies and the transfer of neuroscientific ‘findings’ into the public debate (Vidal, 2005), in particular in the process of teaching and learning. Since gendered conceptions and connotations shape individual actions, social practices, and social segregation, gender-sensitive analyses can help to assess the entanglements within and the outcomes of neuro-pedagogies in medial discourse, schools, and universities. More precisely, such approaches outline the interdependencies of bodily materiality, social experiences, and cultural norms by underpinning the alterability and interdependencies of brains, behavior, thinking, and acting throughout a person’s lifetime. In doing so, they suggest alternative concepts and settings for individual learning processes (e.g., Vidal, 2012; Just, 2014; Mead Vetter, 2014).

## Feminist neurosciences: an outlook—or even a call for further research

Neurofeminist analyses have outlined permanent gender codes in society and in research, such as masculinity, rationality, power, status as subject, to the side of culture, which are contrasted with femininity, emotionality, reproduction, status as object, to the side of nature. The understanding of ‘rationality’ and ‘emotionality’ as gendered material-semiotic nodes (to introduce a term from Haraway (1988)) turns out to be more useful when accounting for their bio-cultural entanglements. In contrast to the prominent and long-standing search for dichotomous differences between women and men, analyses of contextualized differences could be more effective and adequate for realizing gender equity.

Interdisciplinary neurofeminist research on sex/gender neuroscientific knowledge production and on its impacts in current neurocultures should prospectively consider the following aspects: the empirical significance of neuroscientific research, the close entanglements of neuroscientific research with society, and the impacts of ‘neurofacts’ (in the broadest sense) on gendered and intersected cultural symbolisms, social practices, and power relations. Since the embedding of constructive paradigms of ‘neuro-matter-in-society’ is taken as a basis for recent developments surrounding the optimized human within neoliberal framings, the outcomes, vulnerabilities, and power relations of these developments have to be at the core of future discourses on gendered neurocultures (Schmitz, 2014a).

What could these claims mean for critical brain research? The incorporation of ‘dissensus’ questions, as Cynthia Kraus (2012b) suggests, is an important basis for discussing and researching in times of powerful recourses to ‘the neuro’, both in scholarly and public discourse. Although dissensus can be extremely productive for interdisciplinary debates on brain research, Rebecca Jordan-Young (2014) decodes disciplinary language as one reason for the dissensus among neurofeminist scholars. That is, shifting definitions of terms from one discipline to another is fruitful but also bears the danger of misconceptions. A sensitive rereading and negotiation of such conceptual shifts may prevent misleading interpretations. Additionally, scholarly neurofeminism has to further reflect both the use and misuse of its own concepts and how to ask research questions in a study. Based on an “ethic of knowing”, Barad (1996, p. 183) points to a reflection of one’s own research processes because “our constructed knowledges have real material consequences”. Reflective neuroscientific approaches should consider both the situatedness of analyses and the question to what extent scholars’ disciplinary, class, ‘race’, national, and gendered history influence their studies. To what extent are we able to identify the power relations that co-constitute our brains, which in turn influence our research? Based on this individual genealogy, do neurofeminist scholars working on gendered impacts confirm or transform gendered norms and orders through their research?

Finally, the debate on gender-appropriate research must not only take into account neuroscientific knowledge production but also the transformation of neuroscientific ‘facts’ into the public via popular media and pedagogy. Such gendered neurocultures are by no means an exclusive product of separate actors but a result of the entanglements of a multitude of discourses, norms, practices, materialities, and structures that co-constitute each other. In order to rethink brain research today, it is thus all the more important to reflect on these processes in a more detailed manner.

## Conflict of interest statement

The authors declare that the research was conducted in the absence of any commercial or financial relationships that could be construed as a potential conflict of interest.

## Footnotes

^1^This article is a modified version of: Schmitz, S., and Höppner, G. (2014). “Catching the Brain Today: From Neurofeminism to Gendered Neurocultures,” first published in their edited volume *Gendered Neurocultures: Feminist and Queer Perspectives on Current Brain Discourses* (Vienna: Zaglossus). The authors thank their publisher Zaglossus for permission to integrate and reprint parts of the original article.

^2^http://www.genna.gender.uu.se/themes/bodyembodiment/

^3^The following members launched the NeuroGenderings Network: Isabelle Dussauge (Linköping University), Cordelia Fine (University of Melbourne), Hannah Fitsch (Technical University Berlin), Katarina Hamberg (Umeå University), Rebecca Jordan-Young (Columbia University), Anelis Kaiser (University of Bern), Cynthia Kraus (Lausanne University), Emily Ngubia Kuria (Charité Berlin), Katrin Nikoleyczik (University of Freiburg), Deboleena Roy (Emory University), Raffaela Rumiati (International School for Advanced Studies of Trieste), Sigrid Schmitz (University of Vienna), and Catherine Vidal (Institute Pasteur Paris).

^4^For a complete list comprised of over 40 experts who form the current broader NeuroGenderings Network from inter- and transdisciplinary perspectives see: http://neurocultures2012.univie.ac.at/

^5^The program of this ‘dissensus’ conference is available at http://www3.unil.ch/wpmu/neurogenderings3/

^6^http://www.whitehouse.gov/the-press-office/2013/04/02/fact-sheet-brain-initiative

^7^https://www.humanbrainproject.eu/discover/the-project/overview

^8^http://bluebrain.epfl.ch/cms/lang/en/pid/56882
